# HexA is required for growth, aflatoxin biosynthesis and virulence in *Aspergillus flavus*

**DOI:** 10.1186/s12867-019-0121-3

**Published:** 2019-02-11

**Authors:** Jun Yuan, Ding Li, Ling Qin, Jiaojiao Shen, Xiaodong Guo, Elisabeth Tumukunde, Mingzhu Li, Shihua Wang

**Affiliations:** Key Laboratory of Pathogenic Fungi and Mycotoxins of Fujian Province, Key Laboratory of Biopesticide and Chemical Biology of Education Ministry, and School of Life Sciences, Fujian Agriculture and Forestry University, Fuzhou, 350002 China

**Keywords:** *hexA*, *Aspergillus flavus*, Aflatoxins, Virulence

## Abstract

**Background:**

Woronin bodies are fungal-specific organelles whose formation is derived from peroxisomes. The former are believed to be involved in the regulation of mycotoxins biosynthesis, but not in their damage repair function. The hexagonal peroxisome protein (HexA or Hex1) encoded by *hexA* gene in *Aspergillus* is the main and the essential component of the Woronin body. However, little is known about HexA in *Aspergillus flavus*.

**Results:**

In this study, *hexA* knock-out mutant (Δ*hexA*) and complementation strain (Δ*hexA*^C^) were produced using homologous recombination. The results showed that, Δ*hexA* and Δ*hexA*^C^ were successfully constructed. And the data analysis indicated that the colony diameter, stress sensitivity and the sclerotia formation of *A. flavu*s were nearly not affected by the absence of HexA. Yet, the deletion of *hexA* gene reduced the production of asexual spores and lessened virulence on peanuts and maize seeds markedly. In addition, it was also found that there was a significant decrease of Aflatoxin B1 production in deletion mutant, when compared to wild type.

**Conclusions:**

Therefore, it suggested that the *hexA* gene has an essential function in conidia production and secondary metabolism in *A. flavus*. The gene is also believed to be playing an important role in the invasion of *A. flavus* to the host.

## Background

Over the past few years, the food security crisis has been a burning subject all over the world. This problem was not only affecting the human beings’ health, but also closely affected the development of the human society at large. Therefore, a saprophyte fungus *Aspergillus flavus*, was known as a main contaminant of crops. *A. flavus* can infect a variety of major economic crops, such as peanuts, corns, cottonseed and nuts, resulting in extremely serious food safety problems [1–3]. According to a report by the Food and Agriculture Organization of the United Nations, 25% of grain supplies in the world should not be consumed due to mycotoxin contamination which is mainly caused by *A. flavus* [4, 5]. On the other hand, *A. flavus* is the second major cause of invasive aspergillosis in humans [6]. The excess of *A. flavus* spores inhalation or contact with contaminated crops may cause a plurality of parts of the human lung aspergillosis and skin, nails and other infection [7]. These infections are mainly prevalent in areas with high temperatures, such as the Middle East, where the fungus is the most common cause of sinusitis and keratitis [8]. Under appropriate conditions, *A. flavus* will produce a variety of highly toxic and carcinogenic secondary metabolites, such as aflatoxin (AF) and cyclopiazonic acid. The World Health Organization (WHO) has designated aflatoxins as a natural existence of class I carcinogen. Due to stable physical and chemical properties of aflatoxin, simple processing can not get rid of the yellow aspergillus toxin. Therefore, it is very easy to take aflatoxins from contaminated crops into the food chain, resulting in more wide range of contamination and unavoidable economic losses [9].

In order to resist various environmental stresses and injuries from the outside, filamentous fungi developed a set of damage repair mechanisms in the evolution. In 1864, Woronin body in *Ascobolus pulcherrimus* was first described by a Russian scientist Michail Stepanowitsch Woronin [10]. It was then, named by Buller few years later [11]. After that, this organelle was found to be blocking the septal pores in case of damage in a variety of ascomycetes [12–15].

Woronin body has a dense-core consisting of HexA and WSC (woronin sorting complex) [16]. Hence, Hex orthologs in the Woronin body of filamentous fungi played essential roles in the conidial synthesis, fungal resistance [14, 17, 18] and even the fungal virulence [19]. In this study, the homolog of Woronin body core protein found in *A. flavus*, HexA was identified and characterized in order to explore the function of HexA in aflatoxin biosynthesis and development in *A. flavus.*

## Results

### Sequence analysis of HexA in *A. flavus*

**Fig. 1:**
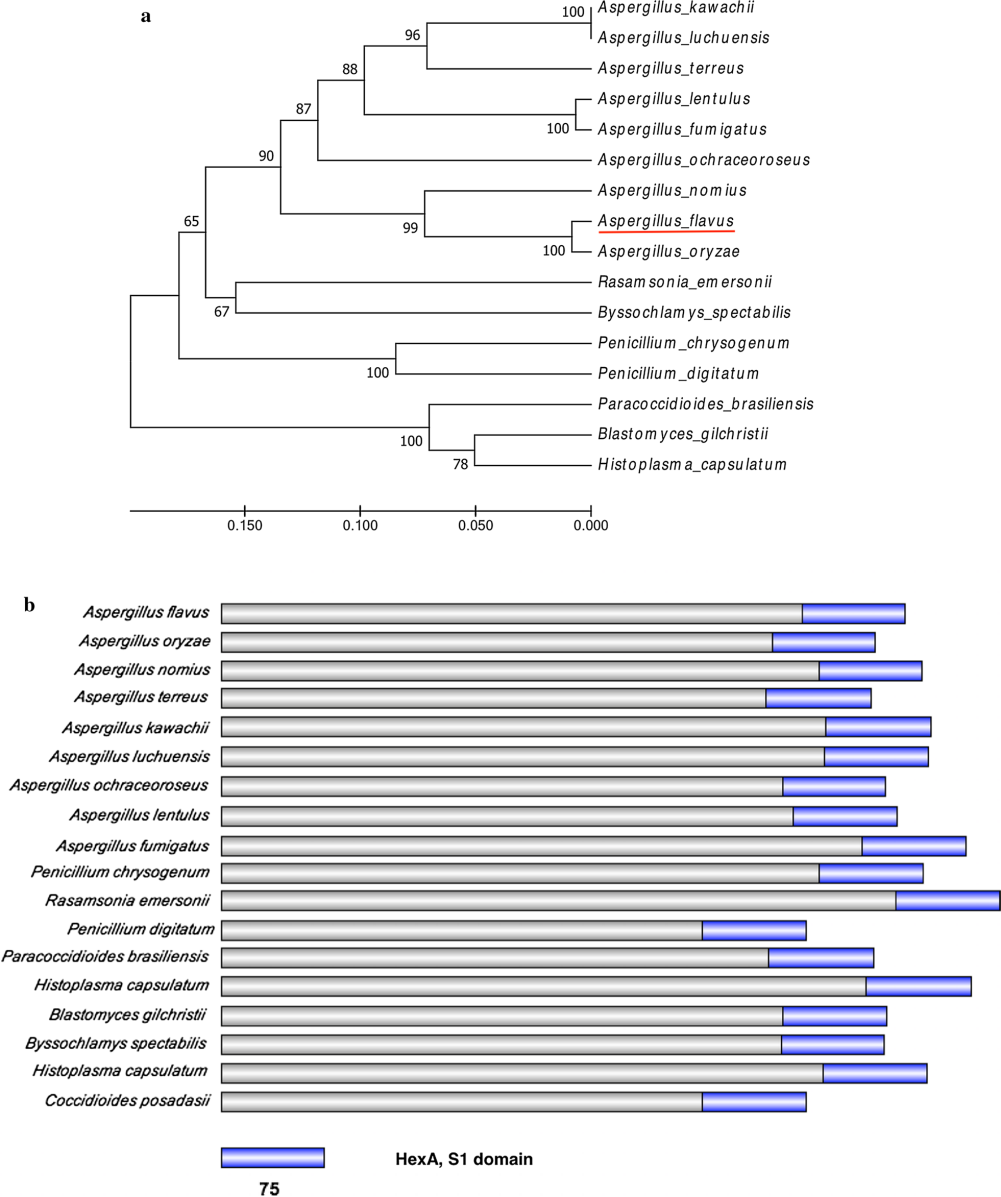
Phylogenetic and structure analysis of HexA proteins. **a** Phylogenetic analysis of HexA proteins from *Aspergillus* members and other fungi. Bootstrap values were calculated using the neighbor-joining method with 1000 replicates. *A. flavus* was underlined in red. **b** Schematic diagrams of HexA proteins. Conserved domain was signed, respectively

HexA protein sequences from different fungi species were downloaded from National Center for Biotechnology Information (NCBI) using “HexA” as the key words. A phylogenetic tree was constructed using the neighbor-joining method by Mega 6.0. It was found that HexA protein was highly conserved in *Aspergillus.* The comparison of amino acid sequences revealed that HexA in *A. flavus* shown the highest identity (83%) to *A. oryzae,* and had the lowest identity (65%) to *A. kawachill and A. luchuensis* (Fig. 1a). Protein sequence analysis demonstrates that HexA protein contains a conserved domain, named S1_Hex1 from 419 to 493 (Fig. 1b). This domain often presented in the HexA protein of fungi suggests that HexA should be a component of Woronin body in *A. flavus*. Meanwhile, it was confirmed that there is only a single copy of the *hexA* gene in the genome by analyzing the published genome sequences of *A. flavus* NRRL3357.

### Generation of *A. flavus* hexA deletion mutants and complementation

**Fig. 2:**
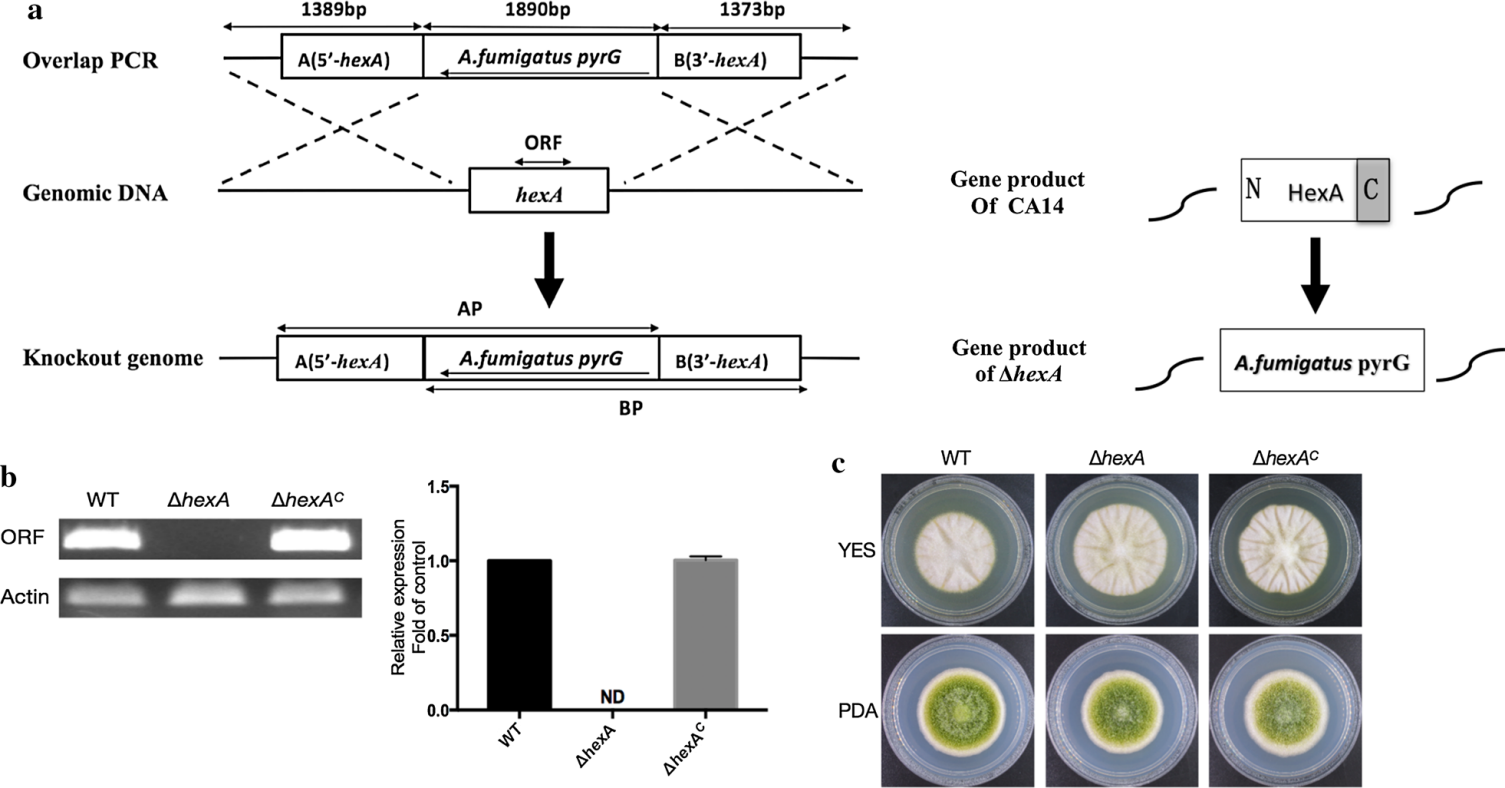
Construction, verification and growth of *hexA* mutants. **a** The scheme of deletion strategy for *hexA*. In the gene product of *hexA,* N terminal (N), C terminal (C) and conserved domain (grey) was signed. **b** q-PCR verification of *hexA* gene deletion and complementation strains. The a*ctin* gene was used as an inner reference. **c** The role of *hexA* gene in mycelium growth

In order to identify the potential biological function of HexA in *A. flavus*, deletion mutants of the *hexA* gene were constructed using pyrG selection. The schematic diagram of homologous recombination strategy is shown in Fig. 2a. Knockout mutant Δ*hexA* was performed using uracil/uridine autotrophy transformants. Afterward, the gene deletion was verified by qPCR with cDNA as a template. The result showed that a 187 bp DNA fragment from the ORF of *hexA* was amplified from WT and Δ*hexA*^C^ by qPCR but not from Δ*hexA* mutant (Fig. 2b). Also, it indicated there was no other homologous gene in the genome. Then the mutant was selected to carry out further study. After inoculated on YES and PDA agar medium for 4 days, the colony phenotypes of the Δ*hexA* mutant was observed. The results showed that there was little difference of colony morphology between the mutant, WT and complementation strains, when they grew on PDA and YES media (Fig. 2c).

### Effects of HexA on the conidia production of *A. flavus*

**Fig. 3:**
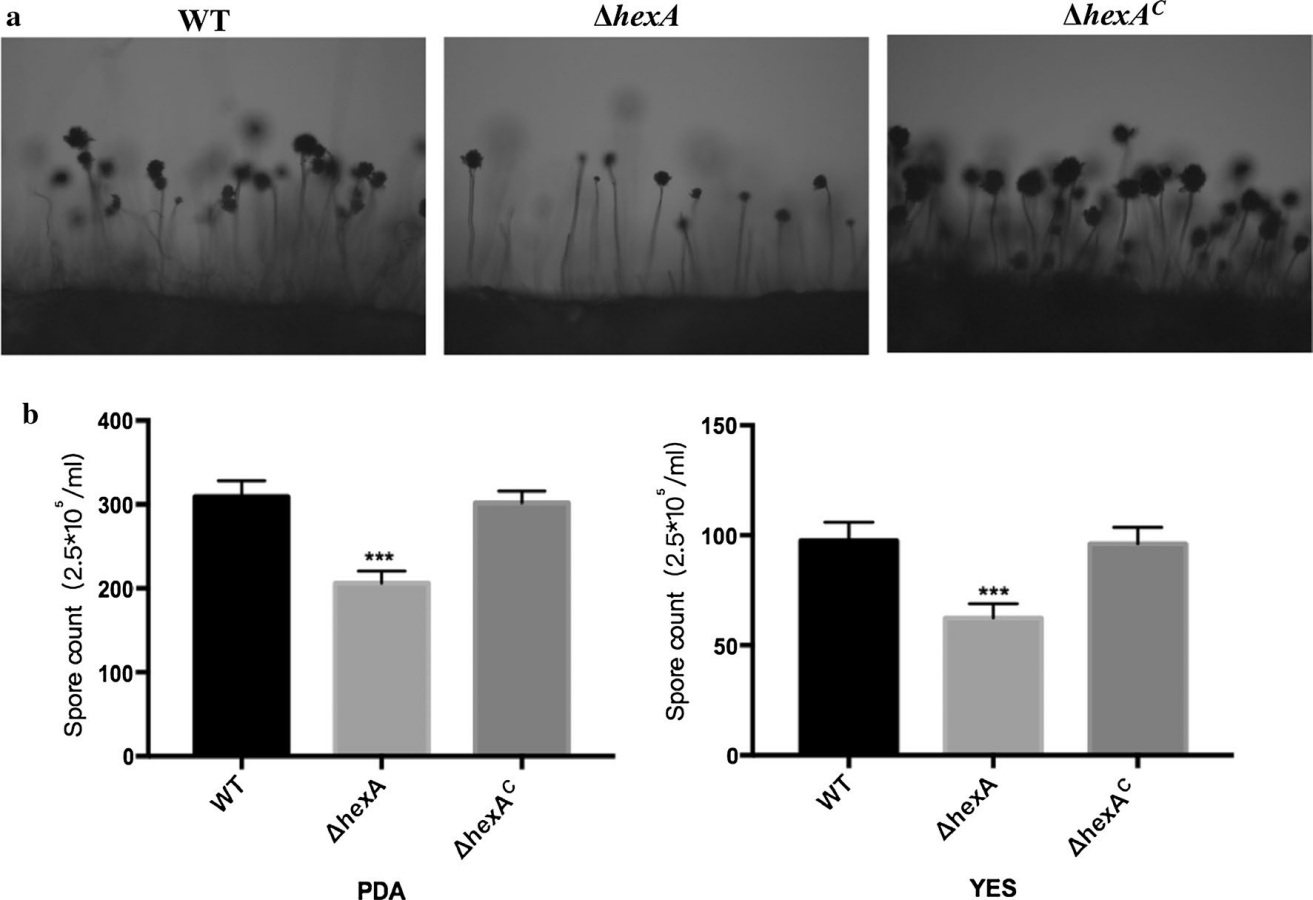
Conidia production is attenuated in null *hexA* mutants. **a** The electron micrograph of conidiophores of WT, Δ*hexA* and Δ*hexA*^C^. Strains were pre-cultured on PDA and Yes were transferred on sterile slides under 37 °C overnight to induce conidiophores. (Magnification scale: ×200) **b** The number of conidia in WT, Δ*hexA* and Δ*hexA*^C^ strains was measured after grown on YES and PDA agar for 5 days at 37 °C. Values are a mean of four replicates. ***Significant difference between the WT and mutant strains at P < 0.001, as assessed by one-way ANOVA and Dunnett’s multiple-comparisons test

After grew on YES and PDA agar medium for 5 days and transfer, the conidiophores of strains were produced and surveyed by electron microscope (Fig. 3a). It was found that in the mutant Δ*hexA*, conidiophores developed imperfectly. The length became much shorter and only a small part of them developed completely. The density and the number of conidia at the top of the conidiophores were significantly decreased. While the conidiophores of WT and Δ*hexA*^C^ was very long, intensive and complete. Also, the data analysis indicated that there was a significant decrease in the conidia production of the knockout strains when compared to the WT or complementation in both the PDA medium and YES medium (Fig. 3b). All these results implied that *hexA* gene might regulate the conidiophore development and the loss of the gene would lead to the reduction of the conidia in *A. flavus*.

### Effects of hexA deletion on sclerotia formation

**Fig. 4:**
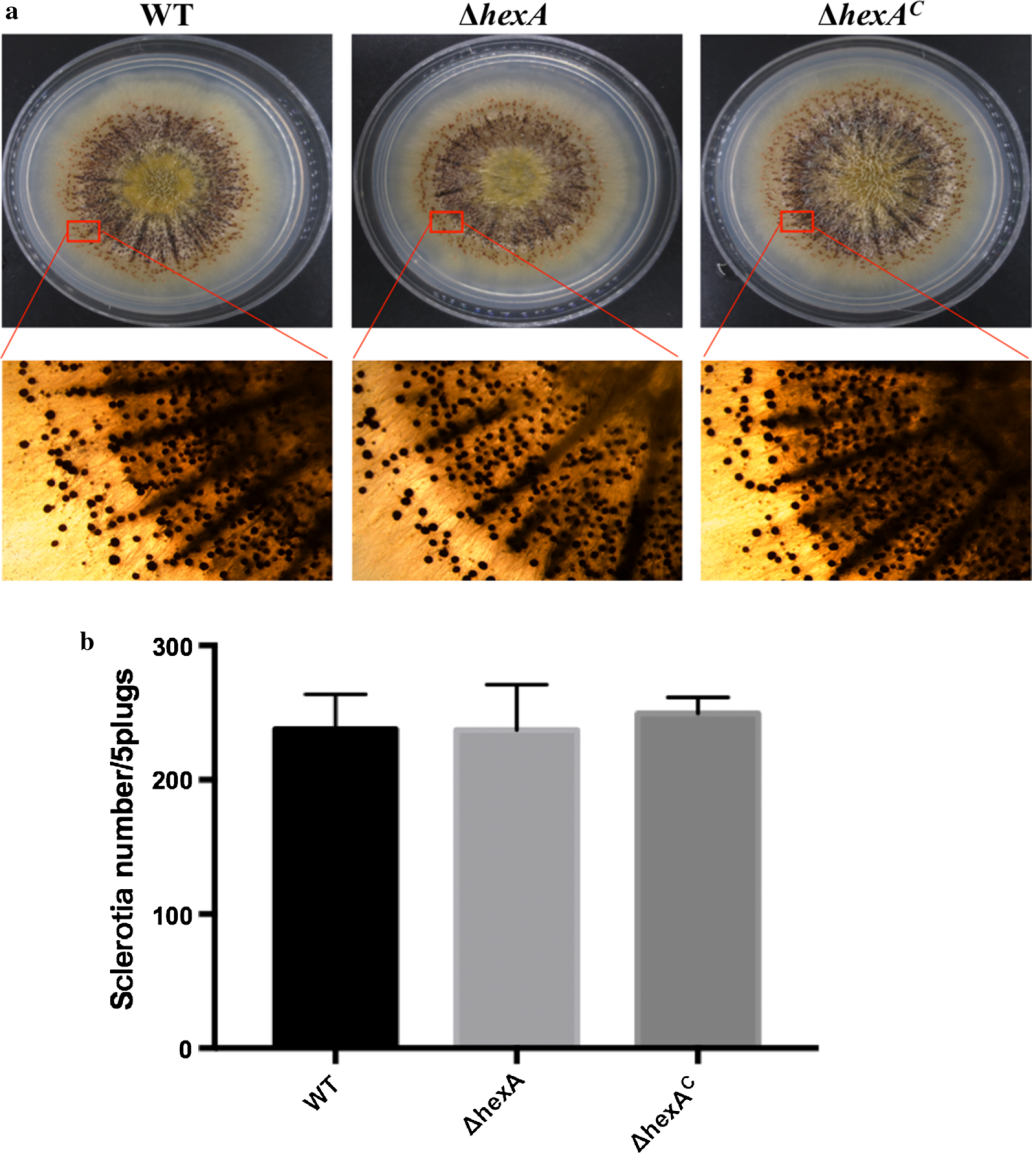
Phenotype analysis of *hexA* deletion, WT and Δ*hexA*^C^ strains on sclerotia production. **a** Morphology of strains on sclerotia-inducing WKM medium after being washed by 75% ethanol and their detail views. **b** The number of sclerotia in WT, Δ*hexA* and Δ*hexA*^C^ strains was measured after grown on WKM medium for 7 days at 37 °C in dark

In order to study whether *hexA* gene was related to the sclerotial production, WT, Δ*hexA* and Δ*hexA*^C^ were inoculated on the sclerotial inducing Wickerham’s solid medium (WKM) at 37 °C for 7 days in dark. The quantity of sclerotium was calculated after washing the media with 75% alcohol. As shown in Fig. 4a, under a microscope, the colony center of the mutant became much thicker than that of WT and Δ*hexA*^C^. And the statistics data indicated there was no significant difference of the sclerotium number between the mutants and WT (P > 0.05) (Fig. 4b).

### Effects of HexA on aflatoxin biosynthesis in *A. flavus*

**Fig. 5:**
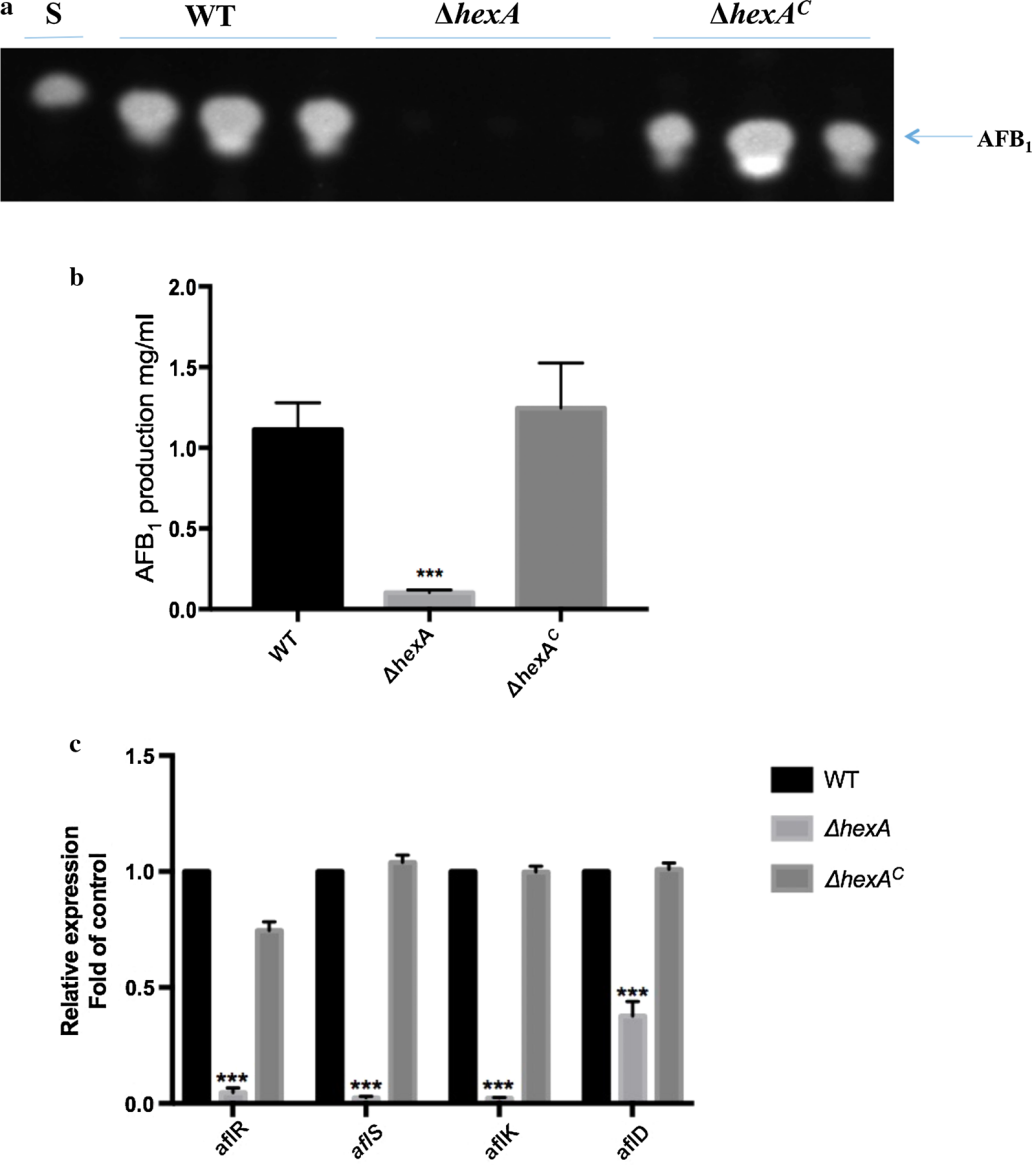
Deletion of *hexA* results in reduced AF production. **a** AFB_1_ production ofWT, Δ*hexA* and Δ*hexA*^C^ were detected by TLC after cultured in YES liquid media for 6 days. **b** Quantification of AFB_1_ production as in **a**. **c** qPCR result of AF biosynthetic related genes (*aflK*, *aflD*, *aflR* and *aflS*) at 72 h. Each bar indicates the mean ± standard deviation (SD) of four replicate assay experiments. ***Significant difference between the WT and mutant strains at P < 0.001, as assessed by one-way ANOVA and Dunnett’s multiple-comparisons test

Aflatoxin is one kind of well-known secondary metabolite of saprophytic fungus *A. flavus*. The production of toxin was associated with the virulence of *A. flavus* to some extent. To determine the effect of *hexA* on AF biosynthesis, thin layer chromatography (TLC) was carried out to test AF production in the WT, Δ*hexA* and Δ*hexA*^C^ after cultivated in liquid YES for 6 days (Fig. 5a). The quantitative analysis results demonstrated that Aflatoxin B_1_ (AFB_1_) in the Δ*hexA* was significantly decreased. The quantitative analysis manifested that amount of AFB_1_ produced by the mutant strains were less than 50% of the WT and Δ*hexA*^C^ (Fig. 5b). Therefore, it indicated that *hexA* gene played an important and positive role in regulating AF production in *A. flavus.*

In order to further verify the function of HexA in modulating secondary metabolite production, and subsequently, the transcription levels of genes that are relevant to aflatoxin biosynthesis were measured by qPCR. In accordance with aflatoxin production, the qPCR results suggested that the loss of *hexA gene* attenuated almost all the transcripts of candidates (Fig. 5c). Among them, included a global transcription factor, *aflR* in the upstream of aflatoxin biosynthesis pathway and a collaborative regulatory gene, *aflS*. Meanwhile, the structure genes in toxin biosynthesis cluster which encoding the key enzymes in the downstream were also detected. By contrast, the expression of *aflK* and *aflD* were severely suppressed compared with the WT and Δ*hexA*^C^. These results showed that HexA might paly an important role in AF biosynthesis by regulating expression of some genes in AF cluster.

### Effects of HexA on stress response of *A. flavus*

**Fig. 6:**
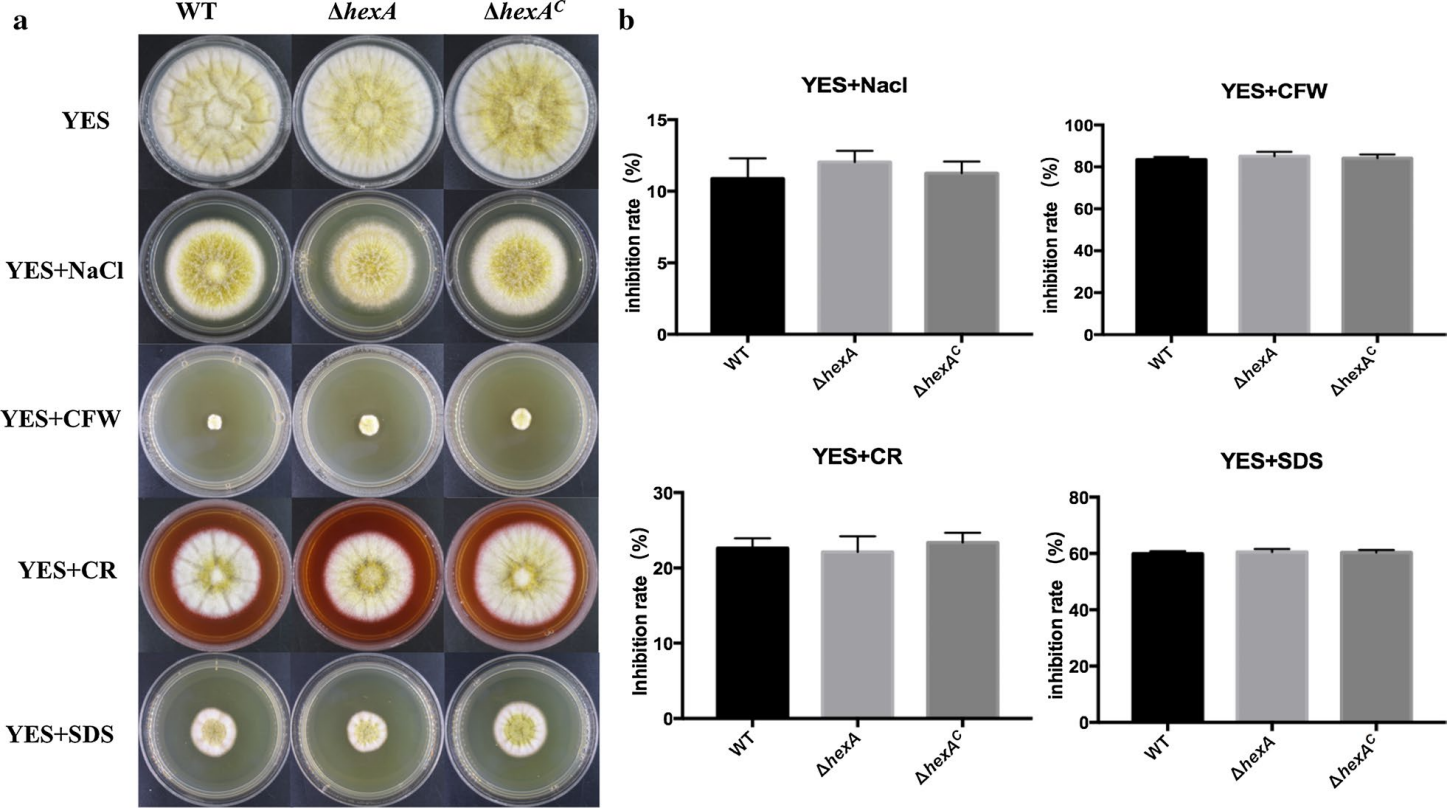
The growth of WT, Δ*hexA* and Δ*hexA*^C^ response to stressors. **a** The strains grew on YES media with 1 M NaCl, 100 μg/mL CFW, 100 μg/mL CR and 100 μg/mL SDS, respectively. **b** Determination of growth inhibition rate of **a**

Woronin body of fungi might play a role in stress resistance of strain according to the previous publications. To this end, several environmental factors such as hyperosmotic stress and cell wall damaging reagent were tested in the study. The morphology of colony was observed after growing up for 5 days (Fig. 6a). Firstly, it could be observed that there was no significant difference in terms of growth between the Δ*hexA* mutant, WT and Δ*hexA*^C^ in the presence of NaCl. Results also suggest that Δ*hexA* mutants were not sensitive to cell-wall damaging agents, Congo red, Calcofluor white and lauryl sodium sulfate (SDS) (Fig. 6a, b). Contrary to previous reports of other fungi [19], these results indicated that HexA was probably not involved in responses to specific stress conditions in *A. flavus*.

### Effects of HexA on pathogenicity to seeds

**Fig. 7:**
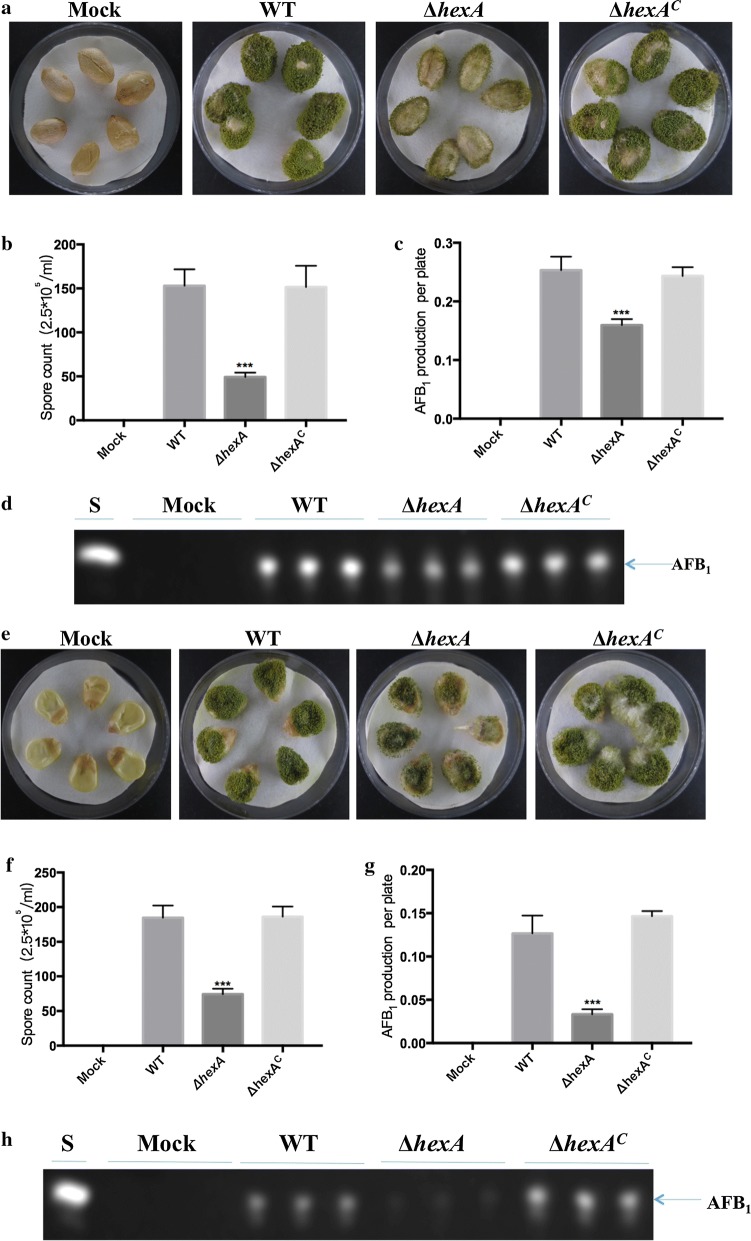
The effect of *hexA* gene on the pathogenicity of WT, Δ*hexA* and Δ*hexA*^C^ of *A. flavus.* **a** The strains of the WT, Δ*hexA* and Δ*hexA*^C^ were grown on peanut seeds at 28 °C for 7 days. **b** Conidia production was assessed from the infected peanut seeds. **c** Quantification of AFB1 production as in **d**. **d** Aflatoxin was detected by TLC, which extracted from infected peanut seeds. **e** The strains of the WT, Δ*hexA* and Δ*hexA*^C^ were grown on maize seeds at 28 °C for 7 days. **f** Conidia production was assessed from the infected maize seeds. **g** Quantification of AFB1 production as in **h**. **h** Aflatoxin was detected by TLC, which extracted from infected maize seeds. ***The bars represent significantly different (P < 0.001)

*Aspergillus flavus* is considered to be one of the most common polluting fungi of the crops. To detect if the deletion of *hexA* might affect seed infections, in this study, the ability of *A. flavus* to infect peanut seeds and maize seeds was tested by colonizing them with Δ*hexA* mutants, WT and Δ*hexA*^C^, respectively. Results indicated that the Mock group had not been infected by *A. flavus*, which showed that the prophase treatment and negative control was successful. At the same time, it was clearly found that skins of peanut and maize seeds have been completely colonized with fluffy mycelium in the WT and Δ*hexA*^C^ Also a large number of deep green conidia were produced in these strains, compared to the Δ*hexA* mutants (Fig. 7a, e). The number of the conidia significantly decreased on peanut and maize seeds inoculated with deletion mutants compared to WT and Δ*hexA*^C^ (Fig. 7b, f). The production of aflatoxin was equally altered in the mutants. Obtained data demonstrated that toxin biosynthesis reduced distinctly in *A. flavus* mutants without *hexA* on peanuts (Fig. 7c, d) and maize seeds (Fig. 7g, h). All these results above suggested that HexA in *A. flavus* took part in the colonization and pathogenicity to the host.

## Discussion

As we all know, a special and unique organelle named “Woronin body” existed in filamentous ascomycete and deuteromycete fungi. It could seal the septal pore when cells countered the cell wall damage factors. According to the researches reported previously, Hex protein was a key part of Woronin body. The *hexA* gene was first found to be responsible for encoding the main component of the Woronin body in *Neurospora crassa* by Jeddand Chua [14]. As a result, Hex protein was given much attention for its quickly repairing function in attenuating the loss of cytoplasm and maintaining cellular integrity. Afterwards, this protein was also identified playing important roles in the other fungi genera in succession, such as *Aspergillus* [20], *Fusarium* [21], *Trichoderma* [22] and *Arthrobotrys* [23]. In this study, we identified the *hexA* gene in *A. flavus,* and bioinformatics analysis indicated that HexA in *A. flavus* was ortholog of Woronin body major protein found and identified in other members of *Aspergillus.* Yuan et al. [15] confirmed that the structural organization of Hex1 in *N. crassa* was similar with eIF-5A in *P. aerophilum* and *Methanococcus jannaschii*. They suggested that *hexA* gene might have been evolved through duplication of an ancestral eIF-5A gene and gained additional features. In the present study, multiple amino acid alignment (data not shown) and conserved region analysis showed that the C-terminal of the HexA proteins in *Aspergillus* was relatively conserved. Similar with *hex1* existed in *N. crassa*, the sequence from 318 to 488 of HexA in *A. flavus* hitted a domain of eukaryotes translation initiation factor eIF-5A, which revealed that HexA was related with eIF-5A in the long-term evolution. But different from the eIF-5A, the sequence of HexA in *A. flavus* possessed a peroxisome target signal site in the C-terminus and a very divergent N-terminus, which suggested that HexA proteins were also related to peroxisomes and might have essential impact on the other aspect of fungal development and physiological activities, except the partial function as eIF-5A.

Ever since the discovery of Hex1 in *N. crassa* [24], several studies have shown that Hex protein was not only an important part of Woronin body conserved in other filamentous fungi systems playing a similar role in controlling the cargo molecules transport from the septal pore. Studies also showed that Hex protein played an essential part in the development and virulence regulation of pathogenic fungi. However, the function performed by HexA in pathogen *A. flavus* is largely unknown. Hence, additional studies on the functions of Hex protein are needed to determine by series experiments. Hence, this study focuses determining the role of *hexA* gene in *A. flavus* in both development modulation and AFs biosynthesis. In *F. graminearum*, the research by Son et al. [21] showed that the lack of Hex1 impaired conidiation and conidial viability. In our study, in two different media (PDA and YES), although there was no clear growth inhibition detected between mutants, WT and Δ*hexA*^C^, it was, however, found that the loss of HexA resulted in the development of defect conidiophores and suppression of conidia production. The above implies that HexA may contribute to cell division and asexual development in *A. flavus*. The similar phenotypes assessed in *A. oligospora,* a famous nematode-trapping fungus. Its mutant strain without *AoHex1* also showed compromised conidiation [23]. The above-indicated studies suggested that HexA was one of the key members in regulating the asexual reproduction in fungi. As one of the most toxic and carcinogenic natural contaminants, AFs are natural polyketide-derived mycotoxins and biosynthesized by an extremely complicated mechanism. Surprisingly, to the best of our knowledge, we, for the first time, found that HexA was related to secondary metabolism in fungi. Our result showed that AFB_1_ biosynthesis was significantly blocked in *ΔhexA* mutants, suggesting that the deletion of *hexA* might result in additional defects and hence affects the aflatoxin production. The qPCR provided further evidence that the absence of HexA down-regulated the expression of genes in aflatoxin biosynthesis cluster indeed. As well as the regulator genes (*aflR* and *aflS*), the transcription levels of *aflK* and *aflD* genes were lowered to some extent. These genes encoded desaturase, and norsolorinic acid ketoreductase respectively, and responsible for the key conversion steps in aflatoxin biosynthesis pathway [25–27]. The deletion of *hexA* depressed the expression of regulatory gene and then modulated the genes in the aflatoxins producing cluster. All these studies indicated that HexA played important roles in regulating the asexual reproduction and exerted a sophisticated effect on the AFB_1_ biosynthesis in *A. flavus*.

Although as reported, there were no clear effects on growth in pathogenic mould *A. fumigatus* under oxidative and hyperosmotic stress in the absence of *hexA*, increased sensitivity were found in the presence of some regents related to integrity of the cell wall and membranes [19]. These results indicated that HexA in *A. fumigatu*s participated in the maintenance of cellular integrity, which was always regarded as basic ability of Woronin body. Interesting, not consistent with the phenomenon above, in our study, no significant changes in sensitivity to stressors such as SDS, Calcofluor white and Congo red were observed between Δ*hexA* mutants and WT. Reports on Woronin body in other fungi showed that except close proximity to septal pores, this organelle sometimes present in nonseptal regions, such as the tips of the germlings and secondary infectious hyphae or at the cell periphery [19, 28–30]. It suggested that the functions of Woronin body and HEX proteins should not be limited as a plug in emergency repair. The research in *Trichoderma atroviride* demonstrated that HEX1 participated in the protection of the cell membrane from dichlorvos stress by reducing excessive electrolyte leakage [31]. In addition, Son et al. reported that Hex proteins might be multifunctional gene. The FgHex1 in *F. graminearum* functioned in the synthesis of both strands of virus genomic RNA. Therefore, we speculated that *hexA* gene in *A. flavus* response to environment stress might function in a different pattern to other species [32].

As we know, several recent studies revealed that Woronin body or Hex protein was also essential in efficient pathogenesis of fungal pathogens [32]. Soundararajan et al. [17] studied the function of Woronin body and Hex1, and found that in the graminaceous-parasitic fungus *Magnaporthe grisea*, no significant difference in morphology or growth rate was determined when Hex1 was absent, compared with the WT and Δ*hexA*^C^. While the infection ability of the strain reduced to about one-fifth for absence of the Woronin body. To verify the pleiotropic effects of Δ*hexA* mutations that appeared on synthetic media, the virulence and toxin contribution of the HexA on peanuts were determined in our research. As a result, the deletion of *hexA* gene significantly decreased the pathogenicity on peanuts, which is in agreement with previous studies in *A. fumigatus* [19] and *M. grisea* [17]. Obtained data indicated that the *hexA* gene played a direct role in the virulence of *A. flavus.*

## Conclusion

In this study, we explored novel function of *hexA* gene in fungal pathogen *A. flavus*. We suggest that HexA can control the asexual processes and contribute to virulence on plant host. What is more, our results diversify the role of HexA, mostly known as a material sealing the septal pore of damaged cell. HexA is required for the production of AFB_1_ by regulating expression of some genes in AF biosynthesis cluster.

## Materials and methods

### Strain and culture conditions

The strain used in the study is listed in Table 1 [33]. *A. flavus* was cultured on YES, WKM or PDA media at 37 °C or 28 °C.

**Table 1:** Wild-type and mutant strains used in this study

Strain	Genotype description	Reference
*A. flavus* CA14PTs	Δ*ku70*, Δ*pyrG*	Nie et al. [33]
Wild-type (WT)	Δ*ku70*	This study
Δ*hexA*	Δ*ku70*, Δ*pyrG*, Δ*hexA*::*pyrG*	This study

### Phylogenetic tree generation

HexA, a protein as a part of Woronin body of *A. flavus* was identified in the NCBI using the blast tool (http://blast.ncbi.nlm.nih.gov/Blast.cgi). The phylogenetic tree was constructed with distinct HexA sequences from different species in *Aspergillus* genus and some other fungi using the Neighbour-joining method by MEGA6.0. Bootstrap analysis was performed with 1000 replicates.

### Construction of ΔhexA mutants and complementation

Homologous recombination was used to generate the HexA mutant. All the primers used in this study were described in Table 2. To construct *hexA* mutants, a 1389 bp DNA fragment upstream from HexA was amplified with *hexA*-A-F and *hexA*-A-R primers. Then, a 1373 bp fragment downstream from HexA was amplified with *hexA*-B-F and *hexA*-B-R primers. P801-R and P1020-F were used to amplify the *pyrG* gene examining for the occurrence of homologous integration in strains. The upstream and downstream fragments with *A. fumigatus pyrG* marker were fused by overlap PCR previously described by Zhuang et al. [34]. To generate the *hexA* mutants, the fusion PCR product was transformed into protoplasts of the CA14 PTSΔ*ku70*Δ*pyrG*. The preparation of protoplasts and transformation was followed the established procedures described by Szewczyk et al. [35] and Yang et al. [36]. For gene complementation, the *hexA* ORF fragment was amplified from *A. flavus* and then transformed into the protoplasts of ∆*hexA* with 2 mg/mL 5-FOA (5-fluoroorotic acid) to replace *pyrG*. After confirmed the removement of *pyrG* geneby PCR analysis was performed to that the *pyrg* gene was removed from the first step of complemented strains. Lastly, the *pyrg* gene was inserted behind the *hexA* to produce the *pyrG* prototroph complementation strains (Δ*hexA*^C^) by using a homologous recombination. The complementation strain was tested using PCR analysis.

**Table 2:** Primers used for the strain construction in this study

Primers	Sequence (5′–3′)	Description
*hexA*-A-F	TCCTCGTAACTTTATCACCG	*hexA* deletion
*hexA*-A-R	GGGTGAAGAGCATTGTTTGAGGCTTCGTTCTTCACGGGTTG	
*hexA*-B-F	GCATCAGTGCCTCCTCTCAGACCTCAACGCCTCGTAAACA	
*hexA*-B-R	GTGGCAGCAAGAGTATGG	
*hexA*-ORF-F	TCCATTCAGCCAGCAACA	
*hexA*-ORF-R	ACACGGGCGTCAAAGTCC	
PyrG/F	GCCTCAAACAATGCTCTTCACCC	*HexA* deletion (*A. fumigatus pyrG*)
PyrG/R	GTCTGAGAGGAGGCACTGATGC	
P801-R	CAGGAGTTCTCGGGTTGTCG	*hexA* mutant screen
P1020-F	ATCGGCAATACCGTCCAGAAGC	
*hexA*-O-F	TGGGTTATGCCTGTTCTGG	*hexA* mutant screen
*hexA*-O-R	GGTTTCTTTCTCGGGTGC	
Step1-F	GGGCAGTAGGTATTGTAGGT	*hexA* complementation
Step1-R	CCTCGGTTCACTACAGCAC	
ORF-UTR-F	TCAAGAGCCTACCTCATACC	
ORF-UTR-R	GGGTGAAGAGCATTGTTTGAGGCACAAGCCACTTCCACCC	
UTR-F	GCATCAGTGCCTCCTCTCAGACTCAACGCCTCGTAAACA	
UTR-R	TGGCAGCAAGAGTATGG	
Overlap-F	AGCCAGTCAACCCACCT	
Overlap-R	CTTACGGCATTAGTACATTTCT	

### Physiology experiments

Conidial suspension (5 mL of a 10^6^ spore/mL) of mutant, WT and complementation strains was inoculated into YES and PDA media, respectively. The colony morphology was observed and the diameter of each colony was recorded after incubation for 4 days at 37 °C. The conidial number was accounted by the haemocytometer after incubation for 5 days at the same temperature in YES and PDA media. At the same time, the strains were transferred on sterile slides under 37 °C overnight to induce conidiophores. Then the conidiophores of strains were produced and surveyed by electron microscope (Magnification scale, 200×). Sclerotium were induced in WKM medium and counted by method described in detail by Yang et al. [36]. For stress resistant analysis, conidia of Δ*hexA*, WT and Δ*hexA*^C^ strains (10^6^ spore/mL) were spotted on YES media added with cell-wall perturbing agent (100 μg/mL Calcofluor white, 100 μg/mL Congo red, 100 μg/mL SDS) or osmotic stress agent (1 M NaCl). All plates were incubated in dark at 37 °C for 5 days, and growth status of colony was observed.

### Pathogenicity tests

To test the infection ability of the strains, pathogenicity tests were carried out by inoculating the kernels of peanuts and maize with WT, Δ*hexA*^C^ and Δ*hexA*. Peanuts and maize were pretreated according to the methods used by Zhang et al. [37]. Then the inoculated kernels were incubated in a plate with 3 layers of wet filter paper at 28 °C for 5 days.

### AF analysis

In order to determinate whether HexA affect the production of AF in *A. flavus*, 10^6^ conidia of each strain were inoculated into 25 mL of liquid YES media in a 100 mL flask shaking at 180 r/m at 28 °C for 6 days. After that, AF was abstracted by chloroform and detected by thin layer chromatography (TLC) and UV light. AFB_1_ standard was purchased from Sigma (Sigma, Germany). The procedures of aflatoxin extraction and detection followed previously described methods [37].

### Real-time fluorescence quantitative reverse transcription PCR (qPCR)

Mycelium of Δ*hexA* mutants, wild-type, and Δ*hexA*^C^ were harvested after incubated for 72 h in YES medium at 28 °C in the dark. RNA was extracted immediately by fully grinding in liquid nitrogen and using the Eastep Total RNA Extraction Kit (Promega, USA). cDNA Synthesis and qPCR was performed using the methods described by Nie et al. [33]. Regulatory genes (*aflR* and *aflS*) and structural genes (*aflK* and *aflD*)were amplified by the primers list in Table 3. *A. flavus* actin gene was used as an endogenous control. The relative quantification of transcripts was calculated by the 2^−∆∆Ct^ method [38]. qPCR assays were conducted with technical triplicates for each sample. And all experiments were repeated twice.

**Table 3:** Primers used for qPCR

Primers	Sequence (5′–3′)	Description
*hexA*/QF	CTCTTCACCCGCCAACT	*hexA* qPCR
*hexA*/QR	CGCCCTGAGGAACAACT	
*aflK*/QF	GAGCGACAGGAGTAACCGTAAG	*aflK* qPCR
*aflK*/QR	CCGATTCCAGACACCATTAGCA	
*aflD*/QF	TGTATGCTCCCGTCCTACTGTTTC	*aflD* qPCR
*aflD*/QR	TGTAGTCTCCTTAGTCGCTTCATC	
*aflS*/QF	CGAGTCGCTCAGGCGCTCAA	*aflS qPCR*
*aflS*/QR	GCTCAGACTGACCGCCGCTC	
*aflR*/*QF*	AAAGCACCCTGTCTTCCCTAAC	*aflR* qPCR
*aflR*/*QR*	GAAGAGGTGGGTCAGTGTTTGTAG	
*actin*/*QF*	ACGGTGTCGTCACAAACTG	*actin* qPCR
*actin*/*QR*	CGGTTGGACTTAGGGTTGATAG	

## Abbreviations

HexA: hexagonal peroxisome protein
AFB_1_: aflatoxin B1
AF: aflatoxin
WT: wild type
WKM: Wickerham’s solid medium
TLC: thin layer chromatography
SDS: lauryl sodium sulfate
